# Dynamic Deposition of PDA on a Hollow Fiber Ceramic Membrane for Oily Water Treatment

**DOI:** 10.1021/acsomega.4c04643

**Published:** 2024-07-26

**Authors:** Bruno da S. G. Alves, Renan F. Barbosa, Ana Clara W. do E. Santo, Alberto C. Habert, Cristiano P. Borges, Fabiana V. da Fonseca

**Affiliations:** † COPPE/Chemical Engineering Program, Federal University of Rio de Janeiro, C. Postal, Rio de Janeiro 21941-972, Brazil; ‡ School of Chemistry, Federal University of Rio de Janeiro, C. Postal, Rio de Janeiro RJ 21941-909, Brazil

## Abstract

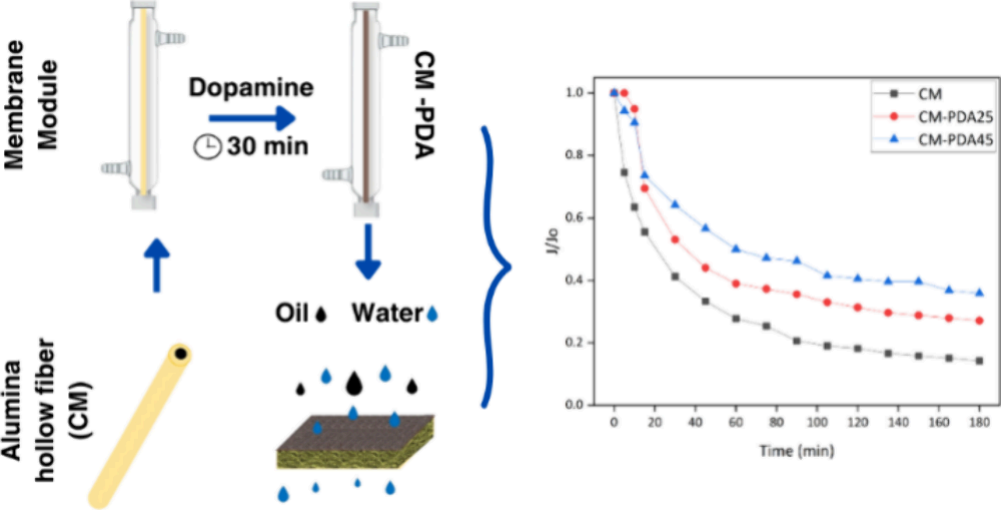

Ceramic membranes have been widely used in oil–water treatment; however, membrane fouling remains a challenge that must be addressed to improve the process feasibility. A thin layer of polydopamine (PDA) was dynamically deposited on the surface of the alumina hollow fiber membranes to reduce oil adhesion. The PDA–alumina membranes were characterized by using SEM-EDS, AFM, and water contact angle measurements. The performance of the modified membranes was evaluated using synthetic crude oil emulsions (100 mg·L^–1^) in a crossflow system. Membranes modified with PDA exhibited 97% oil rejection, and a stabilized permeate flux of 463 L·h^–1^·m^–2^ with a relative flux reduction of 60% and a flux recovery ratio of 75% was observed after cleaning, indicating lower oil adhesion and better fouling reversibility. The most predominant fouling mechanism for the modified membranes seems to be cake filtration because of the reduction in pore size due to the deposition of the PDA layer.

## 1 Introduction

Oily wastewater produced in different industries has become a major concern because it can cause various adverse effects on human health, the environment, and ecological systems if not disposed of properly.^1^ Conventional separation methods for oily wastewater exhibit poor performance for the separation of emulsified oil,^1−3^ and membrane separation is considered one of the most promising technologies in this regard because of its many benefits including high separation efficiency, low energy consumption, and multiple-cycle utilization.^4^ Membrane fouling is a common issue in membrane separation processes and can be attributed to pore blockage, adsorption, concentration polarization, or formation of a gel layer.^5^ This issue can severely compromise the oil–water emulsion treatment process, as it reduces the permeability and cost-effectiveness of the membrane.^6,7^

Membrane fouling decreases permeate flux in constant-pressure systems and raises transmembrane pressure (TMP) in constant-flux systems.^8−10^ Several studies have been conducted in the field of oil–water separation, including research on fouling mechanisms and the improvement of membrane cleaning procedures,^11,12^ as well as investigations into enhancing the hydrophilicity of polymeric membranes to reduce fouling. This enhancement can be achieved through methods such as surface coating with organic or inorganic materials,^13,14^ grafting organic groups,^15^ or incorporating polymers.^16−18^

Compared with polymeric membranes, ceramic membranes exhibit high mechanical strength, a higher separation factor, and a higher flux. However, the high temperatures used in the sintering process to prepare ceramic membranes reduce the number of surface hydroxyl groups, resulting in a membrane surface with fewer hydrophilic groups.^19−21^ This creates a challenge for the large-scale use of ceramic membranes for oil removal owing to their low fouling resistance. Therefore, efforts have been made to develop hydrophilic ceramic membranes with small pore sizes either modifying the ceramic surface by several methods, such as sol–gel, dip-coating, surface grafting, blending or doping, hydrothermal method, and others.

A coating layer of a hydrophilic polymer offers the advantage of flexibility and ease of operation;^20^ however, adhesion between the polymer and ceramic materials remains an important issue.^5,19,22−24^ Furthermore, procedures to enhance the presence of hydroxyl groups on ceramic membrane surface are quite complicated and present challenges for real-world implementation in industrial settings.^25^ One of the simplest alternatives for functionalization is the *in situ* polymerization of polydopamine (PDA), which strongly adheres to various organic and inorganic materials, thereby offering an interesting alternative for surface modification. PDA coating provides a variety of reaction groups (amines, imines, quinones, and catechols) that may facilitate further reactions.^26,27^ Most PDA modifications use polymeric membranes as the substrate and involve the codeposition of dopamine (DA) and additional reactants to promote functional groups at the membrane surface,^28−30^ which can require up to 24 h and may be impractical for industrial use.

Oxidant agents such as permanganate or hydrogen peroxide are used to reduce the time needed for modification of the membrane surface with PDA. Zhu et al.^31^ developed a methodology to accelerate PDA deposition over polyacrylonitrile membranes using iron(III) chloride/hydrogen peroxide (FeCl_3_/H_2_O_2_) under acidic conditions. Fe(III) ions have a high oxidative capacity, increasing the polymerization and deposition rates and forming a stable PDA–PAN composite membrane within 1 h of the reaction. The PDA-modified membranes exhibited a water permeance of 17.5 L·h^–1^·m^–2^·bar^–1^ and dye retention of >98%. Gao et al.^27^ used a solution of CuSO_4_ and H_2_O_2_ to accelerate the deposition of PDA on a ceramic membrane surface and reduce the deposition time. The membrane exhibited superhydrophobicity in air and good stability in several solvents. Wang et al.^22^ formed a 43 nm PDA layer over a flat alumina membrane in just 10 min by introducing KMnO_4_ to oxidize the PDA and improve the polymerization reactivity. The rejection of Congo red by the composite membrane increased from 47.7 to 80.4%, whereas the permeate flux decreased from 524 to 429 L·h^–1^·m^–2^.

Xiong et al.^32^ dynamically deposited a PDA layer under high-temperature conditions in a tubular ceramic membrane to reduce the coating time. This deposition method was performed in a cross-flow filtration system using a solution of DA (1 mg·mL^–1^), CuSO_4_ (5 mM), and H_2_O_2_ (19.6 mM) dissolved at 90 °C in a Tris-HCl buffer solution (pH 8.5, 50 mM). It was used with a cross-flow velocity of 4 m·s^–1^ and transmembrane pressure of 4 bar. The prepared and original membranes exhibited pure water fluxes of 151 and 173 L·h^–1^·m^–2^, respectively. In the evaluation of the antifouling properties of the membrane, it was observed that the prepared membrane demonstrated the highest stable flux for the filtration of brown sugar redissolved syrup.

Although some studies have investigated the modification of ceramic membranes with PDA,^5,22,32,33^ to the best of our knowledge, this is the first work that proposes the dynamic deposition of PDA on hollow fiber ceramic membranes to reduce fouling in oily wastewater. The effect of the temperature on PDA polymerization was investigated to reduce the preparation time. The antifouling performance of the PDA layer in oily wastewater was compared with prior membranes, and a possible fouling mechanism was proposed.

## 2 Results and Discussion

### 2.1 Characterization of Alumina Hollow Fibers

Alumina hollow-fiber microfiltration membranes were prepared for use in oily water treatment, and their surfaces were modified by the dynamic deposition of a thin PDA layer. PDA deposition was expected to reduce the pore size and improve the hydrophilicity of the resulting membrane, which was characterized to better understand the effects of surface modification.

**Figure 1:**
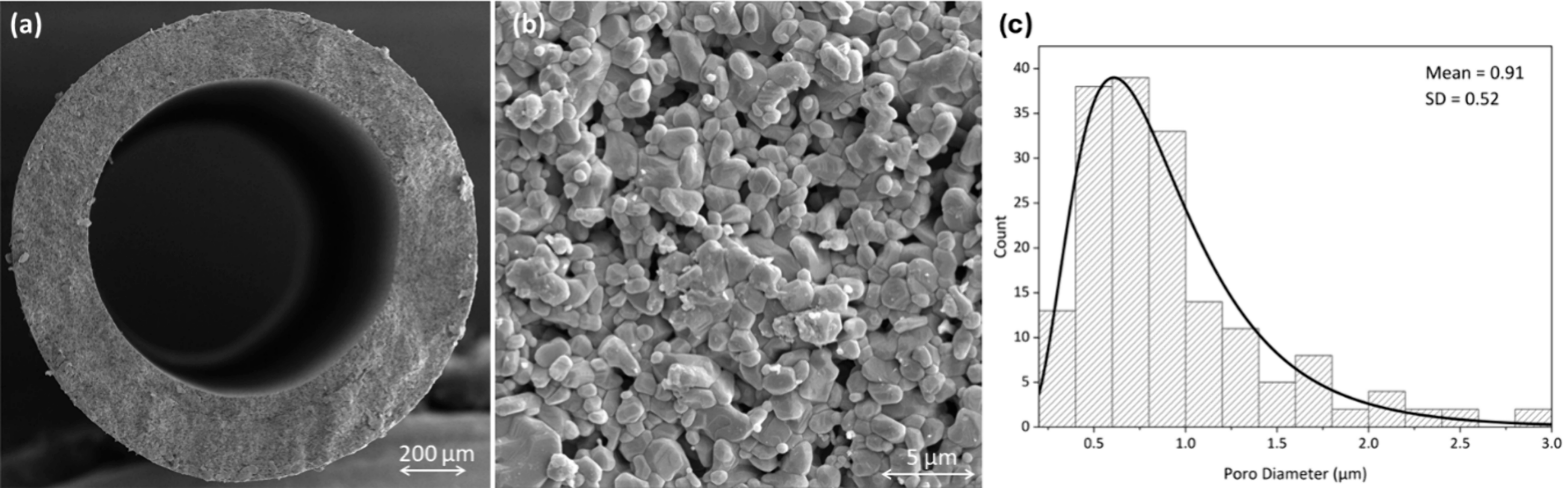
Photomicrographs of the morphology of the alumina hollow fiber membrane: (a) cross section, (b) external surface, and (c) pore diameter distribution.

Figure 1 shows photomicrographs of the morphology of the cross section and the external surface of the alumina hollow fiber. All alumina membranes presented a uniform symmetric sponge-like morphology owing to the fast precipitation rate of the dope suspension and average external and internal diameters of 1.4 and 0.9 mm, respectively. Figure 1b presents the external surface of the hollow fiber, which was used to calculate the mean pore size using the ImageJ software. Pores in ceramic membranes are generally created by voids generated by particle packing; however, the precursor hollow fiber was prepared by the phase inversion process, which induces pore formation by liquid–liquid separation during polymer solution precipitation. Thus, it is expected that the morphology formed by phase inversion remains after polymer degradation by thermal treatment and is fixed by alumina particle sintering. Figure 1c shows the pore diameter distribution of the external surface of the alumina hollow fiber, which had a mean pore diameter of 0.91 μm. The pore distribution analysis shows that the produced membrane has a broad distribution, which is consistent with that of the microfiltration membranes. The large pores did not collapse because of the formation of sintering necks between the larger particles, which was accelerated by increasing the sintering temperature. Similarly, some tiny pores are eliminated because of this phenomenon at high temperatures.^19^ By controlling the heat-treatment conditions and parameters that affect membrane formation through phase inversion, it was possible to produce a porous support with high mechanical resistance and water permeance (2980 L·h^–1^·m^–2^·bar^–1^). The mechanical properties of alumina hollow fiber membranes were characterized by a three-point flexural test, obtaining a flexural stress of 177.5 ± 27 MPa, which is considered adequate for permeation.^19^ The mean pore size and water permeance are compatible with the transport properties of microfiltration membranes; however, usually, for oily water treatment, membranes with smaller pore sizes are selected to achieve high retention and minimize fouling by the pore-blocking mechanism.

### 2.2 Alumina Hollow Fibers Modified by Dynamic Deposition of PDA

**Figure 2:**
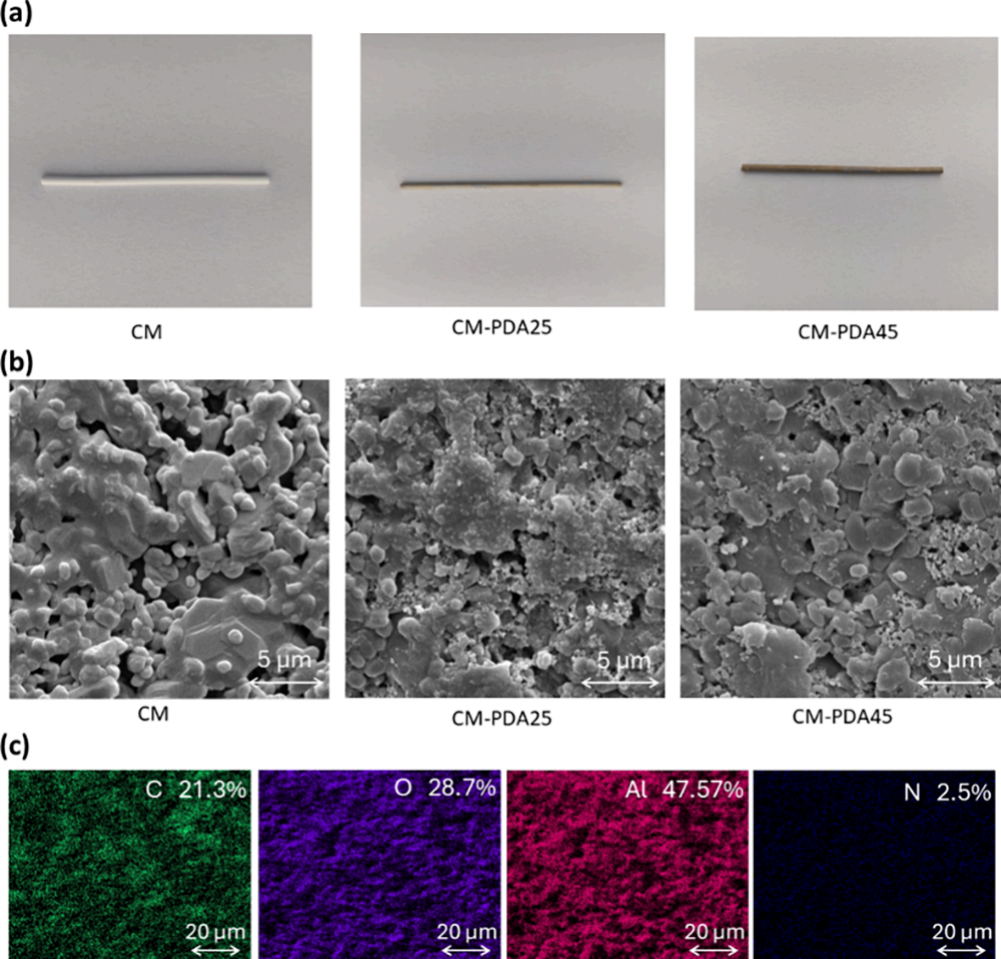
Photographs (a) and SEM photomicrographs (b) of the external surface of pristine (CM) and modified alumina hollow fiber membranes (CM-PDA25 and CM-PDA45); and (c) top surface mapping of the CM-PDA45 with respective atomic percentages.

**Figure 3:**
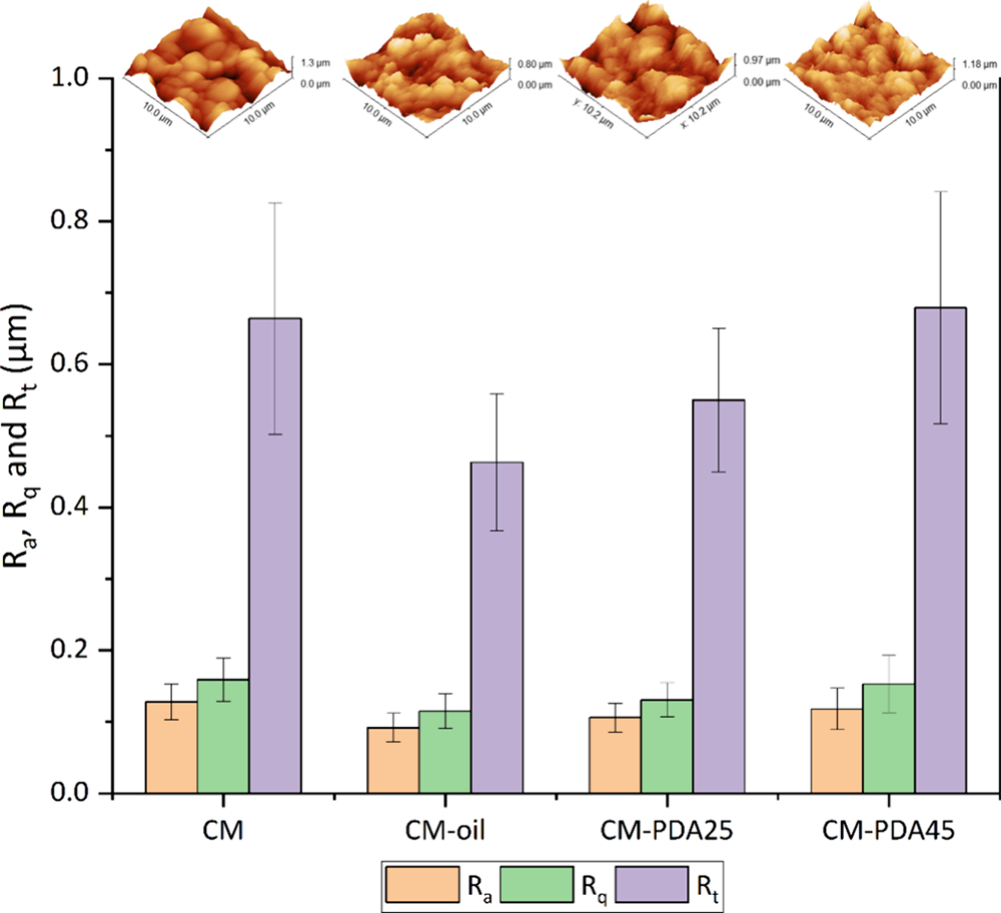
AFM 3D images and roughness parameters (*R*_a_, *R*_q_, and *R*_t_) values of the external surfaces of pristine, pristine fouled (CM-oil), and modified alumina hollow fiber membranes.

The alumina hollow fibers were used to prepare a cross-flow permeation module with oily water being fed into the shell side and permeate collected from the bore side of the fibers. Surface modification with PDA was conducted by recirculation on the feed side of a previously prepared polydopamine (PDA) solution at polymerization temperatures of 25 and 45 °C. Thus, during the recirculation of the solution, PDA was progressively deposited on the alumina surface. The external surface of the alumina hollow fiber membrane before and after PDA deposition was analyzed, and Figures 2 and 3 show SEM photomicrographs and AFM images, respectively.

In Figure 2a, one may note a change in color of the alumina hollow fiber from for CM to brown with PDA deposition; the intensity of brown becomes stronger with the thickness of the PDA layer, as noticed by comparing CM-PDA25 and CM-PDA45, a common phenomenon observed in the deposition of PDA in membranes.^31,32,34,35^ In the photomicrographs in Figure 2b, it is possible to observe the presence of deposited material on the membrane surface, blocking some pores or reducing their effective size. No significant changes were observed in the cross sections of the PDA-modified membranes. In Figure S1, the pore size distributions of these membranes are compared, indicating a reduction in the pore size after PDA deposition. The elemental mapping images in Figure 2c reveal that N is evenly distributed throughout the external surface of the membrane with an atomic ratio of 2.5%, confirming the effectiveness of the PDA coating. The presence of Al (47.5%) and O (28.7%) atoms on the membrane surface is expected for Al_2_O_3_ base membranes.

Figure 3 shows the surface roughness (*R*_a_), root-mean-square roughness (*R*_q_), maximum vertical difference between the highest and lowest points (*R*_t_), and 3D AFM images of the alumina hollow fiber membranes obtained from AFM scans at 10 × 10 μm spots. The brightest area reveals the highest point of the membrane, whereas the dark area presents valleys or membrane pores. It is well-known that membrane surface roughness plays an important role in membrane transport properties because higher surface roughness membranes are related to broad particle distribution and larger pores. In contrast, a smooth surface may reduce deposition on the membrane surface.^36^ The pristine membrane had a surface roughness (*R*_a_ = 0.128 μm and *R*_q_ = 0.159 μm) comparable with results already reported in the literature for alumina hollow fiber membranes for microfiltration.^37^ The modified membranes did not present significant changes in surface roughness compared to the pristine membrane possibly because of the short coating time, thus leading to a thin coating layer. Zhu et al.^31^ observed a decrease in average surface roughness of 22% for coating of PDA onto a surface of a polymeric membrane. Nonetheless, the fouled membrane (after the oil experiment) exhibited a decrease in the surface roughness (*R*_a_ = 0.092 μm and *R*_q_ = 0.115 μm) possibly because of the deposition of oil on the membrane surface.

**Figure 4:**
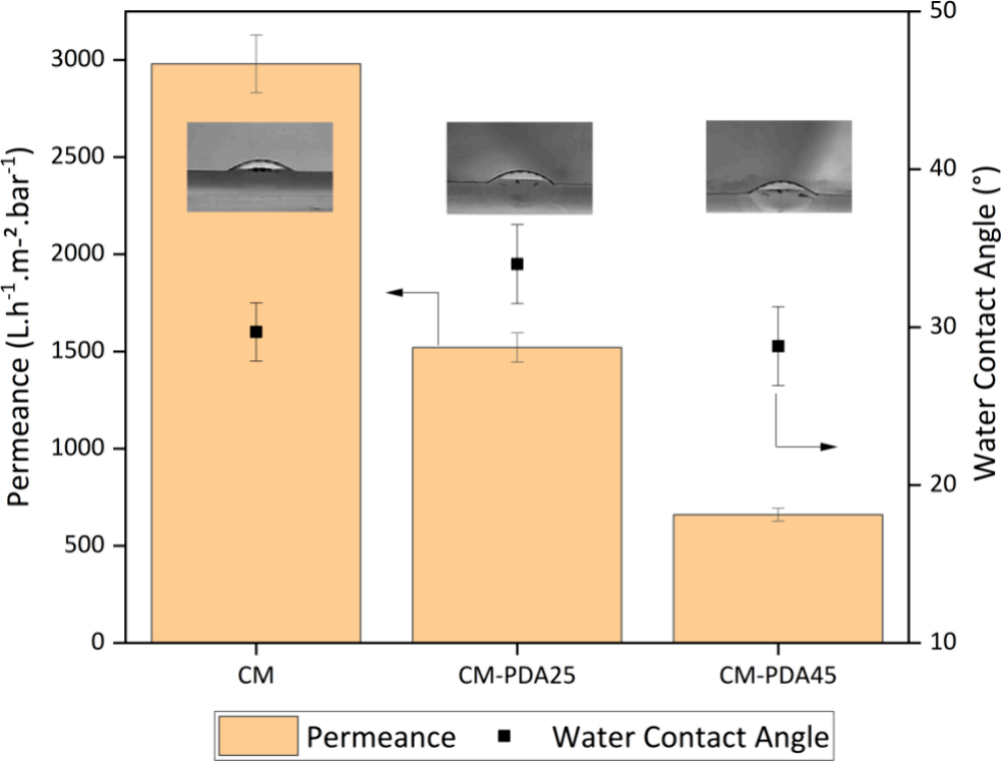
Water permeance and contact angle for pristine and modified alumina hollow fiber.

Pristine and modified alumina hollow fibers were characterized by permeation to determine the water permeance and by measuring the water contact angle to observe changes in the hydrophobicity of the external membrane surface. Figure 4 shows the results obtained for the pristine and PDA-coated membranes. Notably, PDA deposition on the membrane surface reduced the water permeance, which was intensified by PDA prepared at higher polymerization temperatures. These results may be related to the formation of a higher resistance to water permeation owing to the PDA layer blocking the pores of the alumina membrane. The water permeance decreased to 1500 and 660 L·h^–1^·m^–2^·bar^–1^ using the PDA solution after polymerization at 25 and 45 °C, respectively, which could be related to the higher mass weight of PDA at higher polymerization temperatures.

Figure 4 also shows the water contact angles of the pristine and modified membranes after PDA deposition. The unmodified alumina hollow fibers already exhibited a hydrophilic surface, as observed by a water contact angle of 30°. PDA deposition on the external surface of the membrane did not lead to a significant variation in the water contact angle, which was approximately 30° for both PDA deposition conditions, which is also closer to the value observed for a pure PDA film. Hence, coating alumina hollow fibers with PDA reduces the pore size, maintains hydrophilicity, and thereby diminishes the oil adhesion to the surface and pore blocking effect, leading to a decrease in pure water permeance.

### 2.3 Oil–Water Separation

**Figure 5:**
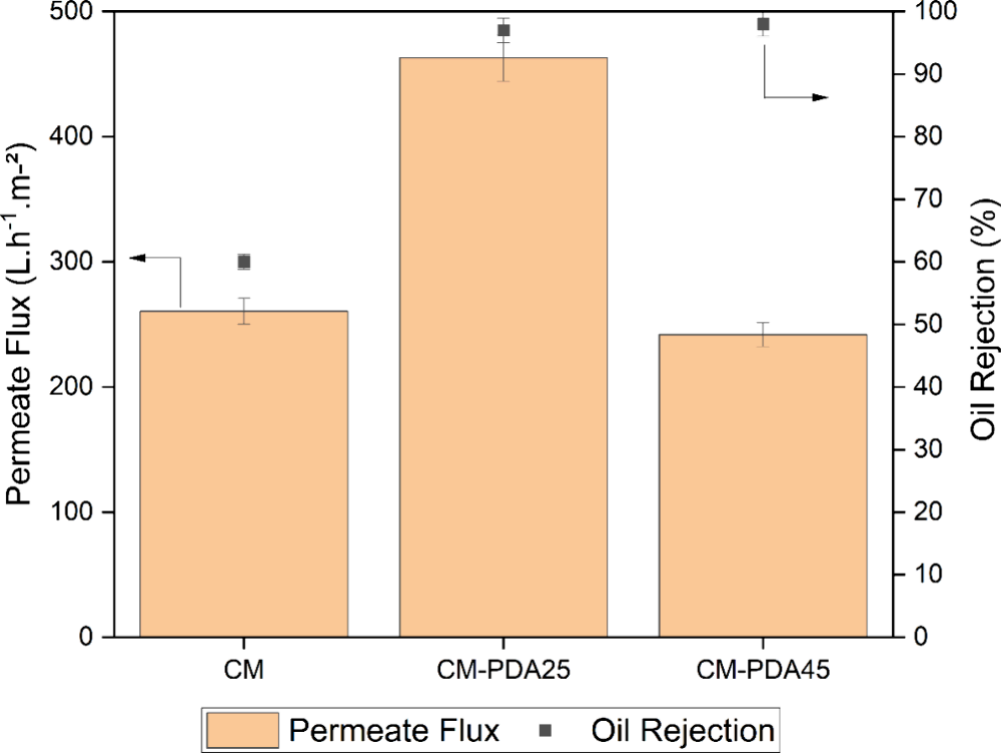
Permeate flux and oil rejection after 180 min of the experiment for the pristine alumina hollow fiber and PDA-coated membranes.

The performance of the membranes for oily water separation was evaluated using an emulsion containing 100 mg·L^–1^ crude oil in water, recirculating the retentate and permeate to the feed tank and periodically measuring permeation flux and oil rejection. Because of membrane fouling, the permeate flux exhibits a continuous reduction over time, approaching an asymptotic value as a function of feed oil concentration, flow regime, and transmembrane pressure difference. For comparison, Figure 5 shows the permeate flux and oil rejection after 180 min of the experiment for the pristine alumina hollow fiber (CM) and for the PDA-coated hollow fibers CM-PDM25 and CM-PDA45.

As discussed previously, the water permeance decreased sharply with PDA deposition on the surface of the alumina hollow fiber, which was related to the partial blocking of the membrane pores. Nevertheless, after 180 min of the experiment, the permeate flux of PDA modified membranes is higher than or equal to the unmodified alumina hollow fiber. CM-PDA25 and CM-PDA45 presented a permeate flux of 463.0 and 241.9 L·h^–1^·m^–2^, respectively, whereas for the unmodified membrane (CM), the value was 260.4 L·h^–1^·m^–2^. These results may be a consequence of the weaker adhesion of the oil phase to the membrane surface, which reduces the extent of membrane fouling. The oil-phase rejection behavior is also interesting: the unmodified membrane had a relatively low oil rejection of 60% because of its larger average pore size. However, after PDA deposition, CM-PDA25 and CM-PDA45 exhibited oil rejections of 97 and 98%, respectively, which may be related to the formation of a PDA layer, leading to higher resistance for oil permeation.

**Figure 6:**
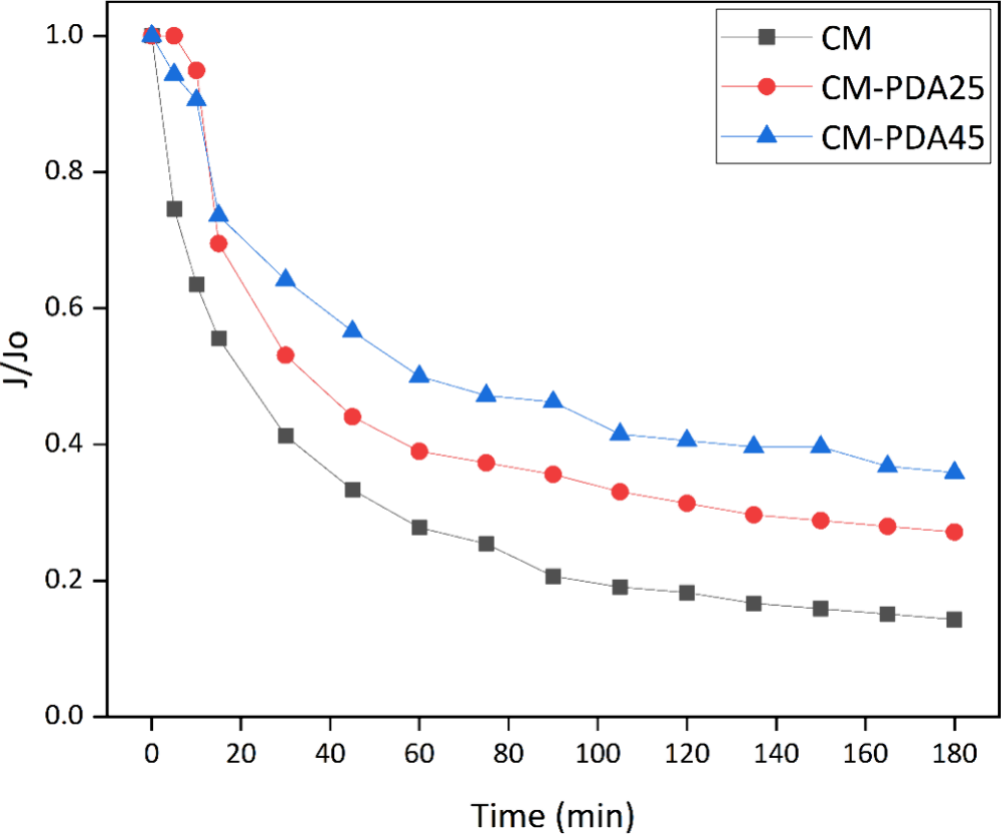
Normalized permeate–flux (*J*/*J*_0_) decline for ceramic and PDA-coated membranes.

The fouling resistance of the PDA modified hollow fiber in comparison with the pristine alumina hollow fiber is better observed in Figure 6, which shows the normalized permeate flux reduction. As shown in Figure 6, the normalized permeate flux (*J*/*J*_o_) of the CM membrane achieved the lowest value (0.14) among the investigated membranes, indicating intensive membrane fouling by the oil phase. On the other hand, in CM-PDA25 and CM-PDA45 membranes, the permeate flux reduction was less intense, achieving values of 0.27 and 0.36, respectively. Despite the fact that different initial water permeate fluxes of each membrane make it difficult to perform an absolute comparison of the membranes, it is possible to infer that a smaller relative flux reduction means a higher fouling resistance. Therefore, the deposition of a PDA layer on the surface of alumina hollow fibers diminishes the interaction between the oil phase and the membrane and reduces the internal pore blockage due to a reduction in the effective pore size at the membrane surface.

**Figure 7:**
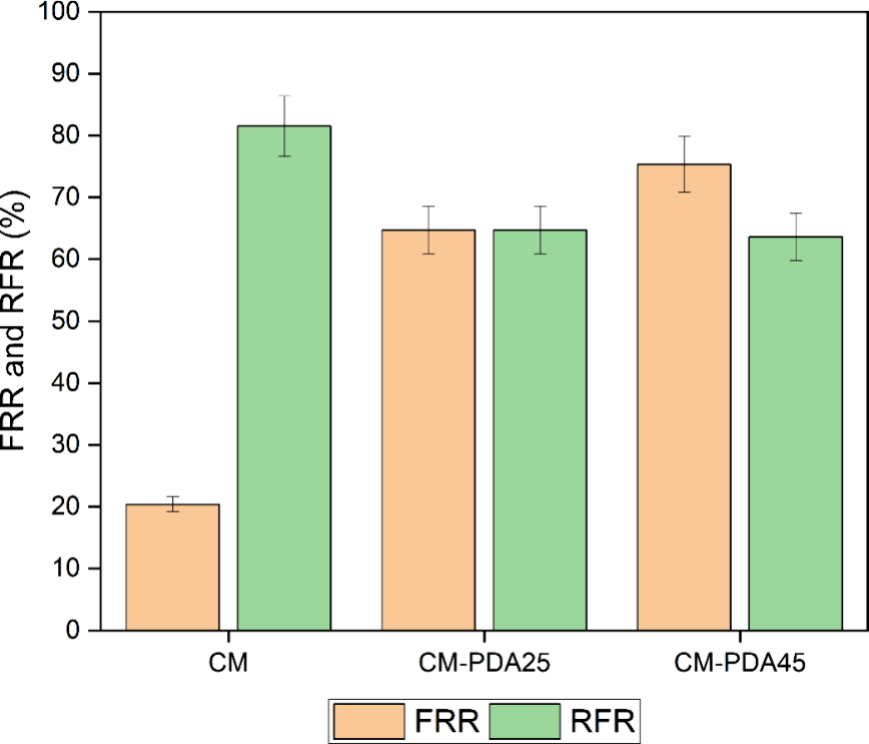
Flux recovery ratio (FRR) and relative flux reduction (RFR) of pristine alumina hollow fiber (CM) and PDA-coated membranes (CM-PDA25 and CM-PSM45).

After the exposure of the membrane to oily water in cross-flow filtration experiments, a layer of foulant was present on the membrane surface, and a reduction in the water flux was expected when compared to the value obtained before contact with the effluent. The relationship between these water fluxes is expressed by the relative flux reduction (RFR, %), defined in eq 4, which indicates the intensity of membrane fouling. As shown in Figure 7, an RFR of 82% was observed for the alumina hollow fiber (CM), whereas for CM-PDA25 and CM-PDA45, it was 64%, confirming less intense deposition of foulant on the membrane when a PDA layer was present.

After oily water permeation experiments, the fouled membranes were subjected to a cleaning procedure by recirculating a 1 g·L^–1^ SDS solution to remove the oil phase that adhered to the membrane. The water permeate flux was measured and compared to the value obtained before contact with the oily water. The relationship between these water fluxes is represented by the flux recovery ratio (FRR, %), defined in eq 3, and it indicates the reversibility of the oil adhesion to the membrane. An FRR as low as 20% was observed for the unmodified alumina hollow fiber (CM), indicating strong fouling of the membrane by the oil-dispersed phase. However, for alumina hollow fiber modified with PDA deposition, it was possible to achieve values of FRR of 65 and 75% for CM-PDA25 and CM-PDA45, respectively. These results clearly demonstrate that the presence of a PDA layer effectively reduces the interaction of alumina with the oil phase, facilitating detachment of deposited oil from the membrane surface.

**Figure 8:**
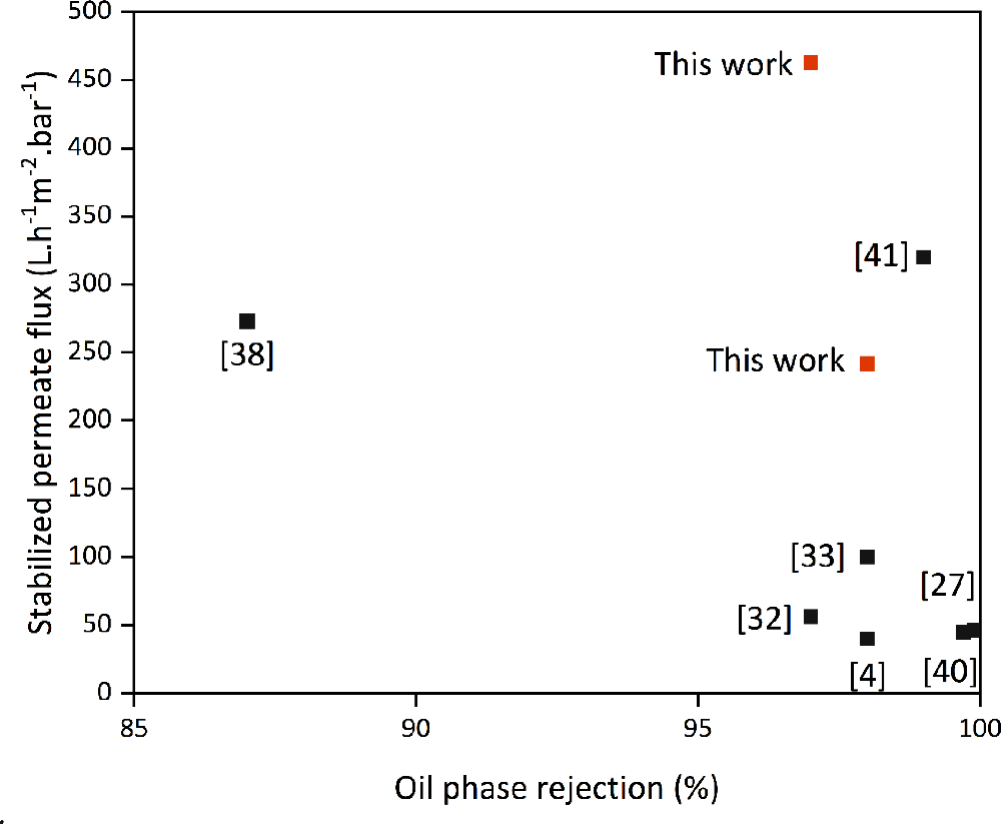
Comparison of stabilized permeate flux and oil rejection of different membranes from the literature and the PDA-coated membranes (CM-PDA25 and CM-PSM45).

Comparison with literature results using different membranes to treat oily water is difficult because of differences in feed composition and concentration, different flow conditions (dead-end and crossflow systems), and different considerations to obtain stabilized permeate flux. Nevertheless, Table 1 and Figure 8 show data from several authors, and for the sake of comparison, some polymer membrane results are also included, allowing one to notice that the PDA-modified alumina hollow fibers exhibit great potential to combine high permeate flux with high rejection of the oily disperse phase. Remarkably, a significant reduction in the initial permeate flux occurred for almost all membranes considered, demonstrating intense membrane fouling during the oily water treatment. Hence, the fouling resistance observed for the PDA-modified alumina hollow fibers is a very important characteristic of process feasibility.

**Table 1:** Comparisons of Ceramic Membranes in Treating an O/W Emulsion

**membrane**	**system**	**clean water permeance(L·h^–1^·m**^**–2**^**·bar**^**–1**^**)**	**feed concentration**(mg·L^–1^)	**rejection (%)**	**stable flux(L·h^–1^·m**^**–2**^**·bar**^**–1**^**)**	**references**
PVDF	crossflow	93	crude oil	97.4	n/a	(38)
PVDF–PDA/PEI	dead-end	3,250	canola oil (1)	98.0	100	(34)
PSf-PDA/KH550/TiO_2_	dead-end	438	crude oil (500)	99.7	45	(30)
ceramic membrane	crossflow	409	crude oil (100)	87.0	273	(39)
Al_2_O_3_/TiO_2_-ZrO_2_	crossflow	917	produced water (100)	N.A.	240	(40)
Al_2_O_3_	crossflow	800	crude oil (250)	99.9	46	(41)
Al_2_O_3_	dead-end	706	crude oil (50)	97.0	56	(42)
Al_2_O_3_-*g*-C_3_N_4_	crossflow	816	crude oil (1000)	99	320	(43)
Al_2_O_3_-PDA/PEI-S	crossflow	272	soybean (500)	>98.0	40	(5)
CM-PDA25	crossflow	1,520	crude oil (100)	97.0	463	this work
CM-PDA45	crossflow	660	crude oil (100)	98.0	242	this work

### 2.4 Fouling Mechanisms

To elucidate the fouling mechanism of both the unmodified alumina hollow fiber and membranes modified with deposition of PDA layer, the experimental data of permeate flux over time were fitted using Hermia’s fouling models. Hermia’s model has been widely used to describe membrane fouling tendency in oily water.^5,30,42,44^ The fouling model can also be used to measure the severity of the deposition of oil on the membrane and to evaluate the improvement achieved by using modified membranes.^45^ The general model equation is as follows:
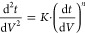
1where *V* (cm^3^) is the permeate volume, *t* is the permeation time, *K* is a phenomenological coefficient for dead-end filtration, and *n* is a general index that assumes different values depending on the fouling mechanism. Pores are completely blocked when the particle size is larger than the membrane pore size (*n* = 2), assuming that particles are larger than the membrane pore size. When the particles are smaller than the membrane pore size, they settle inside the pore walls, leading to internal pore blockage (*n* = 1.5). Intermediate pore blocking occurs when the particle size is in the same range as the membrane pore size and is not necessarily blocked by particles. However, some particles may be deposited on each other (*n* = 1). The last situation occurs when both large and small particles accumulate on the membrane surface to form a cake layer that grows over time (*n* = 0). Table S1 in the Supporting Informationlists the values of *n* and the corresponding linearized equations. The results obtained for each fouling mechanism are summarized in Table 2 and Figure S1.

**Table 2:** Fouling Parameter (*k*) and Data Correlation Coefficient of Hermia’s Filtration Models for the Pristine Alumina Hollow Fiber (CM) and CM-PDA Membranes

**membrane**	**cake filtration**		**standard blocking filtration**		**intermediate blocking filtration**		**complete blockage**	
	***k* (s·m**^**–2**^**)**	***R***^**2**^	***k* (s**^**1/2**^**·m**^**1/2**^**)**	***R***^**2**^	***k* (m**^**–1**^**)**	***R***^**2**^	***k* (s**^**–1**^**)**	***R***^**2**^
CM	1.36 × 10^–09^	0.991	3.06 × 10^–07^	0.986	4.62 × 10^–06^	0.952	2.44 × 10^–04^	0.892
CM-PDA25	4.16 × 10^–10^	0.987	1.50 × 10^–07^	0.933	2.08 × 10^–06^	0.889	1.19 × 10^–04^	0.836
CM-PDA45	1.39 × 10^–09^	0.980	2.43 × 10^–07^	0.941	2.33 × 10^–06^	0.911	9.08 × 10^–05^	0.874

It can be observed in Table 2 and Figure S2 that, for all membranes, the experimental data were best fitted to the cake filtration model, with *R*^2^ values of 0.991, 0.987, and 0.980 for CM, CM-PDA25, and CM-PDA45, respectively. According to Zhang et al.^30^ cake layer formation on the membrane surface resulted from the oil slick, dispersed oil, and emulsified oil. It is noticeable that the intermediate blocking filtration model also was well fitted to data of unmodified alumina hollow fiber, indicating that membrane fouling could be a combination of cake filtration and pore blocking mechanisms, which is expected due to the relatively large pore size (0.91 μm) of this membrane. In the case of CM-PDA25 and CM-PDA45, deposition of the PDA layer reduced the pore size, and the predominant mechanism seems to be cake filtration.

## 3 Conclusions

The surface modification of alumina hollow fiber membranes with polydopamine (PDA) was effective in improving the performance of the membranes for the oily water treatment. PDA deposition on the membrane surface using a dynamic procedure allows the preparation of a modified membrane directly in the permeation module, avoiding several handling steps. The PDA layer on the membrane surface resulted in pore size reduction without significant change in hydrophilicity, which contributed to less oil adhesion to the surface and minimization of pore blockage, as indicated by the permeation results analyzed using Hermia’s fouling model. Although the water permeance was slightly reduced by PDA deposition, the resistance to fouling and oil rejection was significantly improved. Furthermore, the recovery of water permeance after membrane cleaning indicated that the adhesion of oil to the membrane was reversible. Therefore, the surface modification of alumina hollow-fiber membranes with PDA proved to be an effective strategy for enhancing oily water separation, showing significant potential for future applications in treating oil-contaminated waters.

## 4 Methodology

### 4.1 Materials

Commercial alumina powder (Solotest, Brazil) with an average particle size of 4 μm was used to produce the hollow fibers. The dope mixture was then prepared using *N*-methyl-2-pyrrolidinone (NMP, Isofar) as the solvent, poly(ether sulfone) with an average molecular weight of 63,000 g·mol^–1^ (PES, Solvay Veradel 3000P) as the polymeric binder, and polyvinylpyrrolidone (PVP, K90, Sigma-Aldrich) as the dispersant additive. Dopamine hydrochloride (DA) and tris(hydroxymethyl)aminomethane (Tris-HCl) were purchased from Sigma-Aldrich (USA). Sodium dodecyl sulfate (SDS) and hydrochloric acid were purchased from Neon, Ltd. (Brazil). The crude oil (28.1° API gravity) used to prepare the emulsion was supplied by Petrobras, and its physical chemical properties are presented in Table S2.

### 4.2 Preparation of Alumina Hollow Fibers

Alumina microfiltration hollow fiber membranes were produced through phase inversion and sintering based on the methodology used by Barbosa et al.^46^ The dope mixture was formed by dissolving the polymers in a solvent under high-speed mechanical stirring. Alumina powder was gradually added, and the mixture was stirred for 48 h to ensure proper dispersion. This suspension was degassed overnight in a stainless-steel reservoir. After degassing, the spinning suspension was pressurized with nitrogen and extruded through a tube-in-orifice spinneret with specific dimensions (3.8 mm outer diameter and 1.6 mm inner diameter) at a flow rate of 4.0 mL·min^–1^. An air gap of 3.5 cm between the spinneret and the precipitation bath was used. Water was used both in the precipitation bath and as the inner fluid in the fiber bore at a flow rate of 3.0 mL·min^–1^.

**Figure 9:**
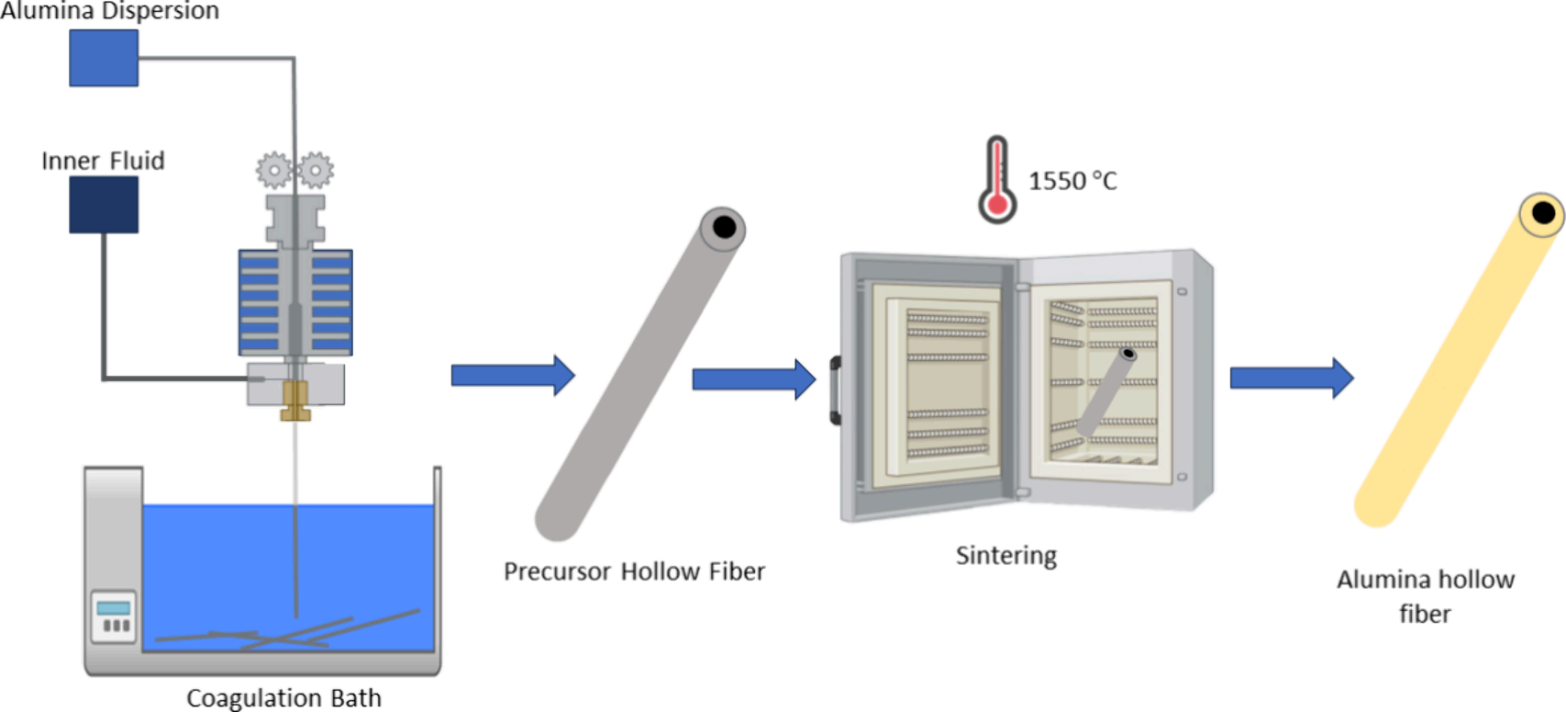
Schematic procedures of steps used for preparation of alumina hollow fiber membranes.

The precursor hollow fibers were immersed in water for 24 h to eliminate the residual solvent. The samples were then cut and air-dried at room temperature. The green fibers were sintered in a tubular electric furnace using a three-stage temperature procedure. Initially, the temperature gradually increased from 25 to 200 °C at a rate of 2 °C.min^–1^ and was held for 1 h. The temperature was then raised to 600 °C at a rate of 2 °C·min^–1^ and held for 2 h. Finally, the temperature was further raised to 1550 °C at a rate of 5 °C·min^–1^ and held for 4 h. The furnace was then cooled slowly to 25 °C. Schematic procedures of steps used for the preparation of alumina hollow fiber membranes are provided in Figure 9. A summary of the spinning and sintering conditions is provided in Table 3.

**Table 3:** Preparation Conditions of Alumina Hollow Fibers

***Dope formulation***	
Al_2_O_3_ (wt %)	45.0
poly(ether sulfone) (wt %)	6.0
polyvinylpyrrolydone (wt %)	0.5
*N*-methylpyrrolydone (wt %)	48.5
***Spinning parameters***	
extrusion rate (mL·min^–1^)	4.0
bore fluid rate (mL·min^–1^)	3.0
air gape (cm)	3.5
internal and external precipitant	microfiltered water
temperature of precipitation	room temperature (ca. 25 °C)
***Sintering parameters***	
sintering temperature (°C)	1,550
heating rate (°C·min^–1^)	2/2/5
temperature plateau (°C)	200/600/1500

### 4.3 Dynamic Deposition of PDA on the Ceramic Hollow Fiber Membrane

**Figure 10:**
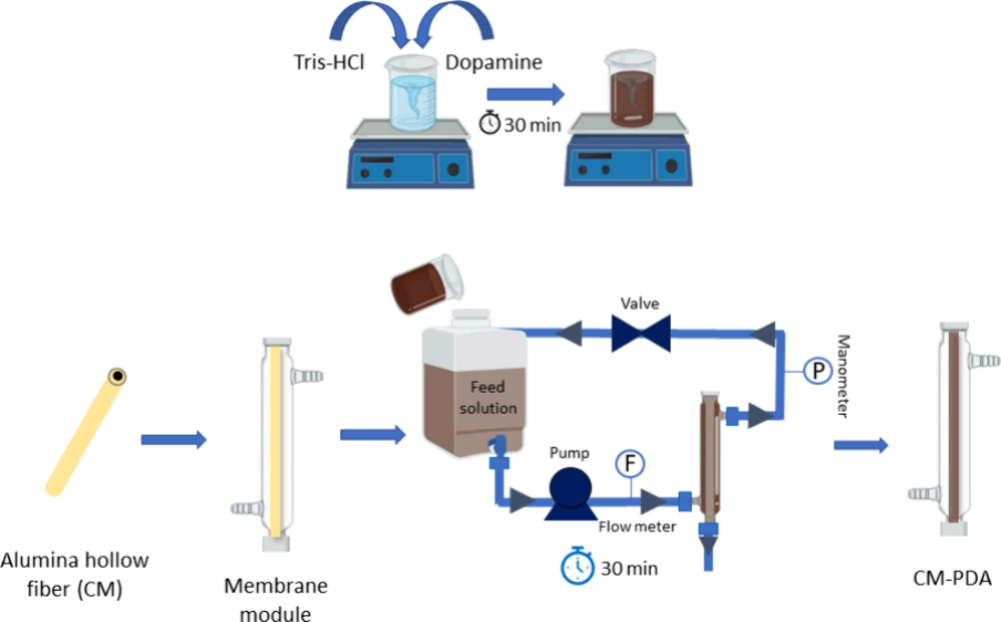
Schematic diagram of the dynamic deposition of PDA on the ceramic hollow fiber membrane.

The experimental device used for dynamic PDA deposition and cross-flow filtration is shown in Figure 10. A PDA functional layer was prepared on the external surface of the alumina hollow fiber membrane by oxidative self-polymerization of dopamine. DA (2 g) was added to 1 L of Tris-HCl buffer solution (pH 8.5, 40 mM) and turned black after 30 min of stirring. Polymerization of DA was conducted at 25 and 45 °C to evaluate the effects of the reaction temperature on the performance of the resulting membranes. The prepared solution was placed in the feed tank and pumped through the membrane module at a flow rate of 125 L·h^–1^ (Reynolds number: 4019). A turbulent flow regime was chosen to reduce the thickness of the PDA layer deposited on the membrane surface and to obtain better uniformity along the fiber length. After 30 min, the system was completely drained and rinsed with deionized water for 2 h to remove loose PDA particles, and the resulting dynamically coated membranes were named CM-PDA25 and CM-PDA45, respectively. During all experimental periods, no delamination or color variation of the modified membrane was observed, indicating that the PDA layer was stable.

### 4.4 Membrane Characterization

The membrane morphology and surface composition were analyzed using scanning electron microscopy (SEM) equipped with energy dispersive X-ray (EDX) (FEI TESCAN VEGA 3, USA). The samples were sputtered with platinum to obtain the membrane images. Atomic force microscopy (AFM, JPK Instruments, Germany) was used to analyze the roughness of the different membranes using the scanning pattern of the probe in the tapping mode, conducted in air, and with a measured area of 10.0 × 10.0 μm. The Gwyddion software was used to analyze images and generate 3D plots. Image analysis was used to calculate the pore size distribution using the ImageJ software, and the SEM images were converted to 8-bit binary (black and white) images, in which the black areas represent the pores and white areas represent the solid surface.^47^ The contact angles (CAs) were measured using the sessile drop method with a water drop of 3 μL. The mechanical properties of alumina hollow fiber membranes were characterized by a texture analyzer (Stable Micro Systems, TA HD plus) with a three-point flexural test with a 0.5 kN load. The error bars represent the standard deviation of at least three measurements.

### 4.5 Measurements of Permeation Performance

The experimental setup is shown in Figure 10. The feed was pumped from the feed tank into the shell side of the membrane module, and permeation occurred from the outside to the inside of the hollow fibers during crossflow filtration. The retentate and permeate streams were then returned to the feed tank. The permeation flux and oil rejection were measured periodically in triplicate using samples from the permeate. The transmembrane pressure (TMP) ranged from 0.3 to 1.2 bar, and the feed flow rate was 125 L·h^–1^ (Reynolds: 4019). The pure water flux was measured under the same conditions. The water permeance was obtained from the angular coefficient of the plot of permeate flux vs TMP. The permeate flux was calculated using eq 1.
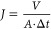
2where *J* is the membrane permeation flux (L·m^–2^·h^–1^), *V* is the volume of the permeate collected in a given time interval (L), *A* is the membrane filtration area (m^2^), and Δ*t* (h) is the operation time interval.

Crude oil was added to distilled water and emulsified using Ultra Turrax (Model T-50) at 11,000 rpm for 10 min. The average oil concentration in the feed stream was 100 mg·L^–1^. During the permeation experiments, the oil concentration was measured in the feed and permeate streams using a dual-beam UV–visible spectrophotometer at 257 nm (UV-2550, Shimadzu, Japan).^14,34,35,42^ The rejection (*R*, %) was calculated using eq 2:
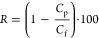
3where *C*_p_ and *C*_f_ are the oil concentrations (mg·L^–1^) in the permeate and feed solutions, respectively. After each experiment with oily water, the membrane was cleaned with 1 L of sodium dodecyl sulfate (SDS, 1 g·L^–1^) solution for 30 min and rinsed with 1 L of deionized water to remove the residual cleaning agent. All permeation experiments and cleaning procedures were conducted without backwashing. To evaluate the antifouling performance, the flux recovery ratio (FRR, %) and relative flux reduction (RFR, %) were calculated using eqs 3 and 4:
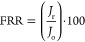
4
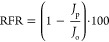
5where *J*_0_ is the pure water flux of the membrane before exposure to oily water, *J*_r_ is the pure water flux of the cleaned membrane after exposure to oily water, and *J*_p_ is the pure water flux after exposition to oily water and before cleaning. The error bars represent the standard deviation of at least three measurements.

## Supporting Information Available

Pore diameter distribution for pristine (CM) and modified alumina hollow fiber membranes (CM-PDA25 and CM-PDA45); correlation coefficient of data fitting using different fouling models of Hermia for pristine alumina hollow fiber (CM) and the PDA coated membranes (CM-PDA25 and CM-PSM45); linearized forms of Hermia’s model for different n values; and oil physical–chemical properties (PDF)
