# Supplementary material for: Dynamic Deposition of PDA on a Hollow Fiber Ceramic Membrane for Oily Water Treatment
Source: ACS Omega. 2024 Jul 26;9(31):34046–55. doi: 10.1021/acsomega.4c04643 (PMC11307986; doi:10.1021/acsomega.4c04643)
Supplement: Supplementary file 1 — ao4c04643_si_001.pdf [file ao4c04643_si_001.pdf]

Supporting Information

Dynamic deposition of PDA on hollow fiber ceramic membrane for oily water treatment

Bruno da S. G. Alves[1*], Renan F. Barbosa[1], Ana Clara W. do E. Santo[2], Alberto C. Habert[1], Cristiano P. Borges[1], Fabiana V. da Fonseca[2]

[1] COPPE/Chemical Engineering Program, Federal University of Rio de Janeiro, C. Postal 68502, 21941-972, Rio de Janeiro, Brazil

[2] School of Chemistry, Federal University of Rio de Janeiro, C. Postal 21941-909, Rio de Janeiro RJ, Brazil

*Email: balves@peq.coppe.ufrj.br

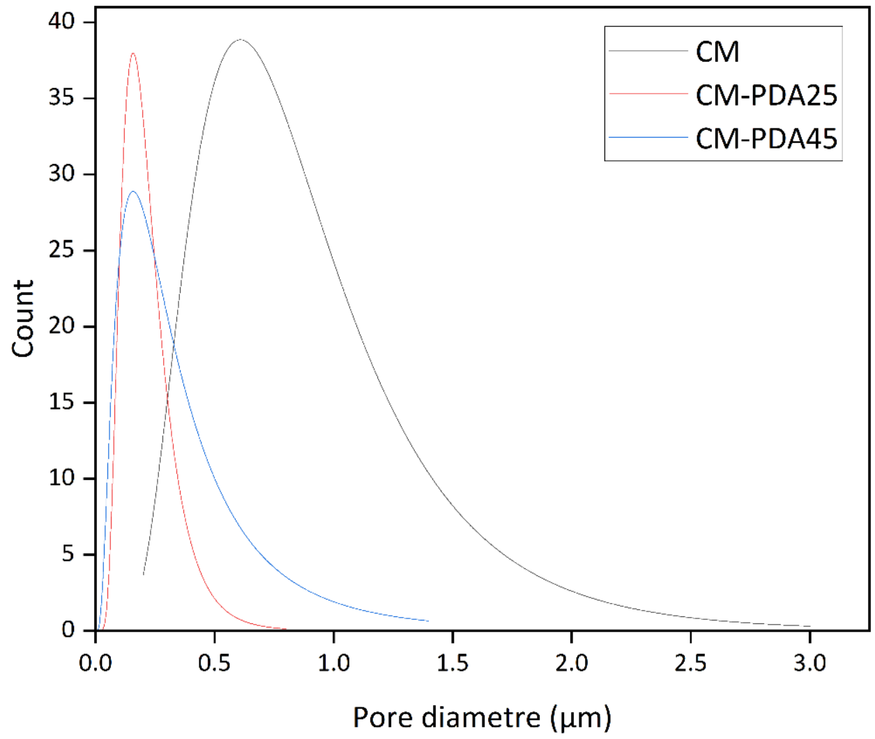

Figure S1. Pore diameter distribution for pristine (CM), and modified alumina hollow fiber membranes (CM-PDA25 and CM-PDA45), with mean values of 0.91 µm, 0.22 µm and 0.40 µm, respectively.

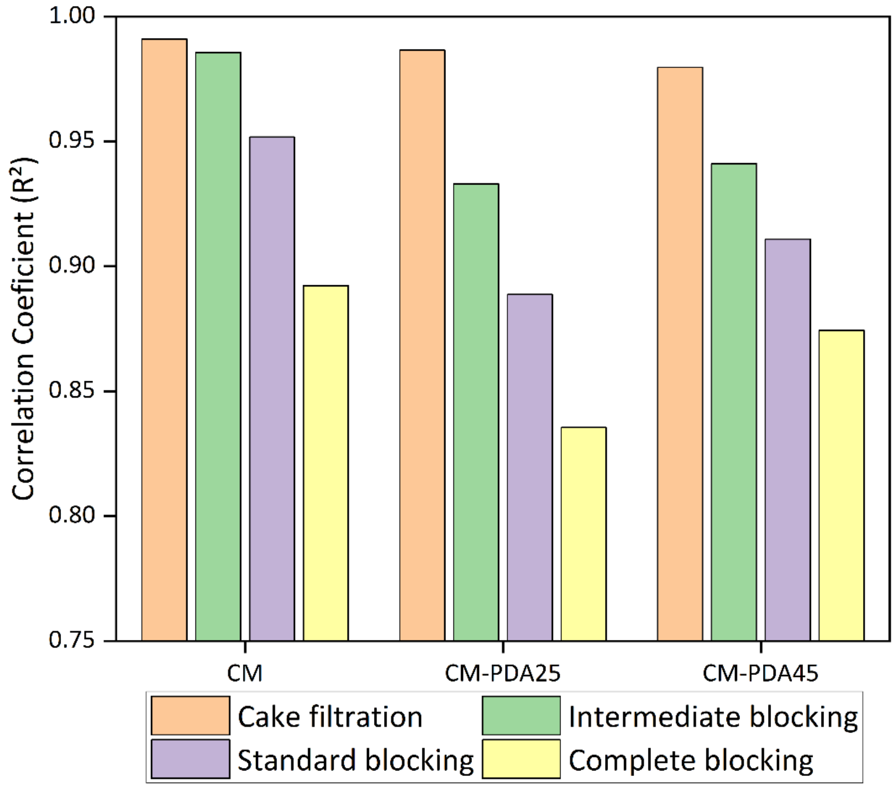

Figure S2. Correlation coefficient of data fitting using different fouling models of Hermia for pristine alumina hollow fiber (CM) and the PDA coated membranes (CM-PDA25 and CM-PSM45).

Table S1. Linearized forms of Hermia's model for different n values.

n	model classification	linearized equation	graphic representation
2	Complete block filtration	$ln\left(\frac{1}{J}\right) = ln\left(\frac{1}{J_o}\right) + K.t$	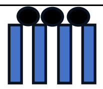
1.5	Standard blocking filtration	$\left(\frac{1}{J}\right)^{1/2} = \left(\frac{1}{J_o}\right)^{1/2} + K.t$	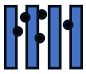
1	Intermediate blocking filtration	$\left(\frac{1}{J}\right) = \left(\frac{1}{J_o}\right) + K.t$	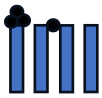
0	Cake Filtration	$\left(\frac{1}{J^2}\right) = \left(\frac{1}{{J_o}^2}\right) + K.t$	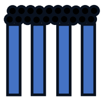

Table S2. Oil physical chemical properties

parameter	unity	value
Density	*°API*	*28.1*
Relative density	*kg/L*	*0.8824*
Pour point	*°C*	*-26*
Viscosity a 25°C	*mPa.s*	*75.0*
Viscosity a 50°C	*mPa.s*	*27.0*
Viscosity a 75°C	*mPa.s*	*13.6*
Water	*%*	*2.9*
Carbon residue	*%wt.*	*4.6*
Asphaltene	*%wt.*	*1.6*
Water (Karl Fisher)	*%*	*0.26*
Resins	*%wt.*	*16.0*

Saturates	*%wt.*	*55.4*
Aromatics	*%wt.*	*27.0*
Watson characterization factor	-	*11,4*
Sulfur	*%wt.*	*0.58*
Nitrogen Total	*%wt.*	*0.30*
Acid total	*mg KOH/g*	*0.09*
Metals	*mg/kg*	
Nickel		*9.0*
Vanadium		*16.0*
